# Balancing the Act

**DOI:** 10.4269/ajtmh.20-1114

**Published:** 2021-02

**Authors:** Madhusudan Samprathi

**Affiliations:** Rainbow Children’s Hospital, Bangalore, India

The morning sunrays shone through the window. The freshly laid out sheets on the bed sparkled in a serene white. The vibrations in the resuscitation room of the pediatric emergency department (ED) infused a fresh wave of energy, inspiring me to make the most of the day ahead. As a fellow in a busy standalone pediatric ED in Northern India which caters annually to about 20,000 patients, I knew this unusual calm and order was in no way predictive of the day that would unfold. My two junior residents walked in with coffee mugs in their hands, their faces fresh and enthusiastic.

As my mind raced through the “to-do” list for the day, a mother’s voice interrupted me. “Is this the pediatric emergency?” Her 10-year-old daughter was unwell for 2 days with a fever. As the nurse started checking her vital signs, I could notice she had a generalized rash. It seemed like a viral fever.

A few other patients had walked in, keeping us occupied. We took turns having our first meal of the day. Unlike the ED, the residents’ lounge reverberated with laughter and camaraderie. Gossip and grumblings poured in from various units. I wondered what they had to grumble about with predictable, orderly, and flexible schedules. I decided to concentrate on my meal, as the next one was uncertain. My meal would determine my decisions and the ever eluding “critical analysis” which was expected of me.

Back in the ED, my residents were busy with more patients. The security staff struggled with relatives crowding in. I scanned the scene to decide which patient to attend first. The resident gave me a quick update. There was a young girl with a neck abscess and septic shock, a teenager with diabetic ketoacidosis and early cerebral edema, and two children with fever. We made a quick plan for the kid with the neck abscess, which included a fluid bolus, first dose antibiotics, reassess, surgery consult, and CT scan once she was stable. We then discussed the plan for the diabetic kid. As I explained the calculations, I wished the referring doctor had performed one simple intervention before referring him as “acute encephalitis”—check his blood sugar. There was a sudden shriek. An anxious lady rushed in with a child, who was having a seizure. The resident and the nurse swung into action and got an IV access. The seizure aborted; it seemed like a febrile seizure.

Before we could realize, it was past lunch time. I ensured that my residents fueled themselves up first. Their work was more tedious. After seeing three children with common cold, two with diarrhea and one with anemia, I contemplated correcting my own hypoglycaemia, when an intubated child was wheeled in. It was a 9-year-old girl with snake envenomation. Her tube appeared displaced. She had been brought in a government ambulance with her father bagging her displaced endotracheal tube, with no accompanying health worker and a crumpled piece of a “referral letter” with the barely legible words—“snake bite.” I quickly moved her in and announced an intubation.

As I gloved up, the nurse told me about another child who “did not seem OK”—a young boy, son of daily wage laborers, found drowsy outside the hutment sometime back. The resident started assessing this boy. I finished the intubation and proceeded toward the boy’s mother to get her story. She looked quite shocked and drowsy herself. Given my own hypoglycemia, I was not much different either. Staring with anxiety and uncertainty, she managed to reveal vague details. I could get no clue to what had transpired. “Will he survive?” she asked me. I wished I knew. I wished he would.

Meanwhile, an angry father was creating a scene, arguing with my resident for being made to wait “too long.” Forced to step in, it took 10 whole precious minutes to placate him. I succeeded. He agreed, to wait longer.

My head was reeling, gut roaring, and legs aching. A teenage boy was wheeled in with a cast in his leg. As I proceeded to see him, my resident snatched the papers from my hand with a smile, “I will see him. You get yourself resuscitated first!” I rushed to grab a quick bite, feeling a pang of guilt to walk past the “angry” father, still waiting to be seen.

Once I was back, the mother of the girl with fever and rash complained that she was vomiting. The girl now looked sick and flushed, and her pulses were bounding. We rushed fluids and started an epinephrine infusion. Guilt loomed. We could have watched her more closely, but when and how was the question.

My phone rang. It was the fellow from the Intensive Care Unit (ICU). There was a bed available. With one bed and four kids needing to go in, the consultant on call would be forced to play God again. Her guilt would probably be greater than mine. I suddenly heard the nurse shout out for help. He had started chest compressions for a new baby, six weeks old, found unresponsive in its sleep. The monitor showed a flat line. We performed cardiopulmonary resuscitation (CPR) for the next 30 minutes. With increasing education and empowerment, it was heartening to see the nurses being efficient team members in the ED, on par with the doctors. The parents were wailing outside. One thing that we will never be habituated to, regardless of how much we do it – is declaring death. Especially to aghast parents of a child, fully well just hours back. I braced myself to look into the father’s eyes. The mother was inconsolable. The father whispered, “Could he have been saved, if we came earlier?” I patted his shoulder. I wished I knew. I wished he could. His guilt was probably more than any of ours. Basic life support training for the general public and bystander CPR are almost unheard of in most low- and middle-income countries (LMICs).

Two new residents arrived with spiral bound workbooks in their hands. Whereas one of them was working on why children are dead on arrival, the other was working on improving the quality of referrals to the ED by training peripheral health workers. It was encouraging to see residents embark on novel, innovative, and relevant research projects to find solutions to day-to-day distressing issues that uniquely plague LMICs.

“Another bed in the ICU,” my night counterpart had arrived, fresh, enthusiastic, and smiling. The child with snake envenomation and the one with diabetic ketoacidosis would get into the ICU. The rest would have to wait. Fortunately, the girl with the rash who had developed shock had stabilized for now. Few specialists see the overwhelming diversity of patients that pediatric emergency physicians do.

I needed some time before I could hand over, to review all the admitted children, to collect test reports, and to collect myself. But there was no time for that, or energy either.

After a long, interrupted hand over, a middle-aged man walked toward me with folded hands. I could recollect his face. He was the father of a young girl who had entered the ED a month back, gasping and pulseless. Today, she smiled at me. She had been fortunate enough to get an ICU bed in 4 hours, and to get oscillated for 4 days. Intensive care had waved its magic wand on her.

As I walked home, the myriad images of the day flashed in my eyes. The wailing parents who lost their baby, the girl who deteriorated “under my eyes,” the blank stare of the drowsy boy’s mother, the angry father who swore at the limitations of our healthcare system, and the father whose eyes welled up with gratitude for his daughter being alive. Each of them had turned to us for support. Admiring the vast sky shimmering with stars, I prayed, “May I be the support rather than the one seeking it.”

Tomorrow would be a new day. A new roller coaster ride. In a place which ceaselessly involves, irritates, overwhelms, teaches, tires, and inspires, all at the same time. I was reminded of a question I was asked in the interview to enter this postdoctoral program. “What essential qualities would a doctor need, to be successful in pediatric critical care?” I had missed one crucial word–balance. To not just survive, but learn and grow through the roller coaster. I yearned for the morning sunrays and the ED vibrations that would recharge me again.

